# Books and Magazines

**Published:** 1913-05

**Authors:** 


### Books and Magazines.

Solidified Carbon Dioxide: The Successful Treatment of Cutaneous Neoplasms and Other Skin Diseases, With Special Reference to Angioma, Epithelioma and Lupus Erythematosus. Fully illustrated. By Ralph Bernstein, M. D., Philadelphia, clinical instructor in skin diseases, Hahnemann Medical College, Philadelphia; consulting dermatologist to the Women's Southern Homeopathic Hospital, Philadelphia; consulting dermatologist to the J. Lewis Crozier Hospital and Home for Incurables, Chester, Pa., etc., etc., etc. Frank S. Betz Company, Hammond, Ind. Price not stated.

This strikes me as being a very valuable handbook on skin diseases and the use of a new remedy in their treatment. If the solidified carbon dioxide will do for others one-half that it has done for Dr. Bernstein, the remedy is practically as specific and is invaluable. He cures epithelioma with it, something nobody else ever did with anything, unless I except the cancer cure quacks who burn it out. The claims made for the remedy by Dr. Bernstein are almost incredible, yet his reputation entitles them to respect. There is a great deal in the little book besides the clinical report of cases, illustrated; for instance: The origin of C. D. as a therapeutic measure. Liquid air. Ethyl-chloride, general considerations, instructions in the technique; method of solidifying; preservation, apparatus, crayons. It is really a thesaurus to the dermatologist and I see no reason why, with this book for a guide—the country doctor as well as the specialist, should not use the remedy successfully in everyday practice. Price not stated, but it is not over $1, I should say. It is splendidly illustrated.

---

Outlines of Physiology. By Edward Groves Jones, A. B., M. D., professor of surgery in Atlanta School of Medicine; and Allen H. Bunce, A. B., M. D., associate professor of physiology in same school. Third edition, revised, 111 illustrations. Price not stated. Philadelphia, P. Blakiston's Son & Co., 1912.

Several chapters have been added to this edition, viz.: "The Cell," 'The Elementary Tissues" and "The Blood," also the Physiological Characteristics of Muscle, and new matter has been added to the physiology of Digestion and Absorption. It is a handbook really, on the relation of physiology to the practice of medicine.

---

Skin Grafting, for Surgeons and General Practitioners. By Leonard Freeman, B. S., M. A., M. D., professor of surgery Medical Department University of Colorado, surgeon to St. Joseph's Hospital, etc., Denver, Colo., 24 illustrations. C. V. Mosby Company, St. Louis, 1912. Price $1.50.

Dr. Freeman goes pretty fully into the subject and presents in neat and compact form the history of skin-grafting from the earliest attempts by the ancient Hindus. He brings it down through the ages, to date, giving some historic cases; dwells upon the necessity of surgical cleanliness; gives, chapter 3, Reverdin's method, which, in December, 1869, startled the world, he says, by its audacity, now universally followed; tells the reader how to prepare for the operation, method of cutting the grafts, etc., applying them, dressing, etc. Chapter IV gives in detail, very imporant, especially to the novice, Thieroch's method. This is a valuable manual and can be read and referred to with profit by even the most expert grafters. It is all about grafting, skin, bone, tissue, etc.

---

Diseases of the Heart and Aorta. By Arthur Douglass Hirschfelder, M. D., associate in medicine, Johns Hopkins University, with an introductory note by Lewellyn F. Barker, M. D., LL. D., professor of Medicine, Johns Hopkins University. 344 illustrations by the author; second edition. J. B. Lippincott Company, Philadelphia and London. Price, $6.

The domain of medicine is so broad and constantly widening that in order to keep abreast of the progress in the many departments it is necessary that comprehensive monographs shall be written, supplementary to the text books on general medicine and surgery, and recognizing the want of a definite work on the heart and aorta, Dr. Hirschfelder has given to the profession an elaborate treatise on the subject, copiously and beautifully illustrated by drawings by the author. The first edition appeared some time ago. We have before us a beautiful second edition. Since the appearance of the first edition, numerous advances have been made in the subject of cardiac disease of such importance that, in order to embody them in the text, several chapters had to be rewritten and enlarged. The electro-cardiograph, which has now assumed a role of primary importance in physiology, pharmacology and clinical observation is in this edition discussed in detail as to theory, construction and application. This instrument has thrown much light on the mechanism of the normal and pathological heart beats, as was to be expected, and is in many clinics a routine practice. The general practitioner of medicine should lose no time in making himself acquainted with the advances in this important branch of study. The section on the pathology of arteriosclerosis has been rewritten, the investigations upon syphilitic arterial disease having made possible a clearer classification of that diseases.

Diseases of Women: A Clinical Guide to Their Diagnosis and Treatment. By George Ernest Herman, M. D., London, F. R. C. P., consulting physician to the London Hospital; late president of the Obstetrical Society of London, etc. Enlarged edition, revised by the author, assisted by R. Drummond Maxwell, M. D., London, F. R. C. S. Eng., assistant obstetric physician to the London Hospital, etc. Illustrated with eight full-page color plates and 292 figures in the text. Octavo; price, $7.50, net. Funk & Wagnalls Company, New York, publishers.

Here is a magnificent work, by one of the greatest living obstetricians. It is an extensive, all inclusive and thoroughly reliable clinical guide to the diagnosis and treatment of diseases of women. The author, in a brief preface, says:

"I have written this book because it seemed to me that a book was wanted which should guide the student and practitioner to the diagnosis and right treatment of the diseases of women. I have tried to present the different diseases which affect the organs peculiar to women, in the way in which they appear in practice. I have not sought to quote everything that has been recommended, but to state principles, and in applying those principles to recommend that which, having tried it, I know to be good. I may plead that I have one qualification for writing such a book, not always possessed by the authors of the text-books—namely, clinical experience.

---

Building a Profitable Practice: Being a Text-Book on Medical Economics. By Thomas F. Reilly, M. D., professor of applied therapeutics, medical department Fordham University, New York City. J. B. Lippincott Company, Philadelphia and London, 1913. Price not stated.

Since the practice of medicine is no longer a "mission," (and a fee,—"honorarium"), but a calling, a vocation, an occupation, by which thousands make a living and some a fortune, it becomes necessary that the pupils (I had nearly said "pupa") should be instructed in "how to do it"; and this book meets my views and the exigencies of the case, exactly. To succeed financially in the practice of medicine requires something more than a knowledge of medicine, however thorough. Those requirements are detailed by Dr. Reilly, and I cannot go into it here. I will only say that I have seen a medical scholar burn the midnight oil, in vain,—and a fellow open up next door to him and burn no oil whatever, yet take all the bread and butter away from him. The scholar did not have the *savoir faire*—the other fellow did. The biggest fool I ever knew was a profound Greek scholar.

